# Mechanisms influencing Chinese senior faculty’s intention to engage in volunteer teaching and policy implications

**DOI:** 10.3389/fpsyg.2026.1834256

**Published:** 2026-05-21

**Authors:** Shuo Ma, Zhiqing Zhang, Qi Yuan

**Affiliations:** School of Economics and Management, Nanjing Tech University, Nanjing, China

**Keywords:** norm activation model, silver-age teacher, structural equation modeling, theory of planned behavior, volunteer teaching intention

## Abstract

As China’s population continues to age, effectively harnessing the human resources of its elderly population has become a critical strategic direction for the nation’s medium-to-long-term development. Against this backdrop, college and university silver-age teachers – retired senior intellectuals from institutions of higher education with extensive experience in teaching and research – represent high-quality intellectual resources. Their participation and sustained dedication to voluntary teaching initiatives are pivotal to unlocking the potential of senior talents and advancing the balanced development of education. Current policy practices face a central puzzle: Why do some silver-age teachers choose to continue contributing in recipient areas after their terms end, while others depart? To uncover the psychological factors underlying this phenomenon, this study integrates Norm Activation Model (NAM) with the Theory of Planned Behavior (TPB). Using respondent-driven sampling to collect data from university silver-age teachers participating in the program, we employ structural equation modeling (SEM) to validate variable pathways. The aim is to elucidate the intrinsic mechanisms driving their initial participation and long-term retention intentions. Results indicate that personal norms serve as the core mediating variable driving volunteer teaching commitment, exerting a significant positive effect on volunteer teaching intention. Problem perception, outcome efficacy, and subjective norms all indirectly enhance teaching commitment by reinforcing personal norms, with outcome efficacy significantly influencing subjective norms. This study reveals the transmission logic of “external cognition-norm internalization-behavioral intention,” providing theoretical and practical references for optimizing senior volunteer teaching policies, planning teacher volunteer pathways, and constructing interactive ecosystems for recipient communities.

## 1 Introduction

Population aging has become an irreversible national reality in China’s medium-to-long-term development process, with its profound impact on social resource allocation, public service provision, and human resource development increasingly evident. Data from China’s National Bureau of Statistics indicates that the proportion of the population aged 65 and above has entered a phase of rapid ascent. Research projections suggest this ratio will exceed 25% around 2030, marking China’s formal entry into a deeply aged society (ESCAP, 2022). Globally, demographic transition has become a shared challenge. The United Nations’ 2023 World Social Report calls on nations to adopt proactive, healthy, and inclusive aging strategies, advocating measures such as social participation, lifelong learning, and flexible employment to fully unleash the potential of older populations and promote sustainable social development (Wilmoth et al., 2023). Within this framework, policies such as delaying retirement, encouraging re-employment of older adults, and promoting volunteer services have become key options for many countries to address labor shortages and foster intergenerational integration.

Against this backdrop, in 2023, the General Offices of three central ministries – the Ministry of Education, the Ministry of Finance, and the Ministry of Human Resources and Social Security of the People’s Republic of China – jointly issued the National Silver-Age Teachers Action Plan. This initiative aims to systematically organize, support, and encourage outstanding retired teachers to re-engage in education. By means of voluntary teaching, lectures, teaching and research, and other forms, it channels their extensive teaching experience and professional knowledge into weak links and much-needed areas across basic education, vocational education, and higher education. The launch of this plan has upgraded silver-age teachers’ voluntary teaching efforts from local pilot programs to a national-level systematic strategy, clearly establishing the strategic direction of tapping into retired teachers’ resources and facilitating the high-quality development of education. It marks a new stage of standardized and institutionalized development in the development and utilization of China’s silver-age teacher resources.

Since the implementation of the plan, positive results have been achieved in promoting the balanced allocation of educational resources and leveraging the professional value of retired teachers. However, a critical bottleneck remains in practice: there are disparities in the stability of silver-age teachers’ participation. Some teachers choose to renew their terms and stay rooted in frontline voluntary teaching after their tenure ends, while others opt to leave. This reality of coexisting retention and attrition reflects that the formation mechanism of teachers’ intention to engage in voluntary teaching has not yet been fully uncovered. Specifically, existing supportive measures mostly focus on material security and personnel deployment, with insufficient attention paid to the psychological motivations, social support mechanisms and emotional demands behind teachers’ participation willingness; existing studies also mostly remain at the level of policy interpretation or descriptive analysis of pilot outcomes, failing to explain why teachers make drastically different behavioral choices after participation.

To deeply analyze the sustained participation mechanism and the root causes of behavioral differentiation among silver-age teachers during volunteer teaching, this study integrates two classic theoretical frameworks: the Norm Activation Model and the Theory of Planned Behavior. The Norm Activation Model was first proposed by Schwartz (1977). Centered on explaining moral altruistic behavior, this theory emphasizes that individuals’ cognitive judgment of public problems and sense of responsibility activate internal personal norms, which in turn drive prosocial decisions and voluntary behavioral choices. With a mature theoretical system and stable explanatory power, it remains widely applied in research on public-welfare behaviors such as volunteer services and educational assistance (Nketiah et al., 2022; Majeed et al., 2023). The Theory of Planned Behavior was systematically refined by Ajzen (1991). From the perspective of rational action, this theory focuses on the intrinsic links among subjective norms, behavioral cognition, and intention, to systematically explain the decision logic and driving pathways of individuals’ voluntary participation in public services, public-welfare aid, and other voluntary behaviors (Lee et al., 2023; Pillai et al., 2022). Combining the complementary strengths of the two theories, this study constructs a multidimensional theoretical model encompassing problem perception, outcome efficacy, subjective norms, personal norms, and volunteer teaching intention, and adopts structural equation modeling for quantitative testing. The core paradigm of this statistical method was established by Bollen (1989), which can effectively avoid measurement bias of latent variables and accurately identify complex pathways, mediating transmission, and effect strengths among multiple variables. It has become a core analytical tool for empirical research in social behavior and education (Savari et al., 2023; Ding et al., 2023). Based on questionnaire sample data, this study systematically verifies the pathway relationships and mediating mechanisms among all variables. The results reveal that personal norms play a core mediating role in the formation of volunteer teaching intention. Problem perception, outcome efficacy, and subjective norms indirectly influence intention by strengthening personal norms.

Theoretically, this study fills the research gap in the interdisciplinary field of the psychological mechanism of elderly prosocial behavior and educational resource allocation, and uncovers the internal psychological path of silver-age teachers from participation to retention. Practically, it provides a mechanistic explanation for understanding the behavioral differences between teachers who stay rooted and those who leave, and offers empirical evidence for the targeted optimization of the National Silver-Age Teachers Action Plan, the differentiated design of teacher support policies, and the construction of talent retention mechanisms in recipient regions. In doing so, it provides solid support for realizing the sustained release of silver-age talent potential, advancing educational equity, and actively responding to the national strategy of population aging.

## 2 Literature review and research hypotheses

### 2.1 The influence of problem awareness on personal norms

According to the core proposition of the Norm Activation Model (NAM), problem awareness is a critical prerequisite for activating personal norms (Schwartz, 1977; Fu, 2024). In the decision-making context of silver-age teachers participating in voluntary teaching, when teachers gain a profound understanding of the practical predicaments of backward educational quality and a shortage of high-quality teachers in central and western China as well as remote areas through policy publicity, field visits, peer exchanges or other channels, and recognize that such educational imbalance hinders the achievement of educational equity, their professional mission as educators and intrinsic motivation to make the most of their senior years will be significantly stimulated.

Specifically, when silver-age teachers realize that their inaction may exacerbate educational imbalance and exert negative impacts on the development of students in recipient regions, their internal moral responsibility will be aroused, thereby generating a sense of internal accountability. This cognition-responsibility transformation mechanism is particularly prominent in the context of educational support. As Confente and Scarpi (2021) pointed out that individuals’ awareness of adverse environmental consequences serves as a key prerequisite for stimulating environmentally responsible behavior among both tourists and residents. Similarly, Song et al. (2023) confirmed in the field of sustainable consumption that consumers’ awareness of packaging waste issues directly and positively affects their moral obligation to adopt eco-friendly packaging. In the context of silver-age teachers, a profound cognition of educational imbalance will also be transformed into an intrinsic imperative to engage in voluntary teaching, prompting teachers to internalize the support for western education from external advocacy into personal moral responsibility.

Based on this, this study proposes the following hypothesis:

H1: Problem awareness has a positive effect on personal norms.

### 2.2 The influence of outcome efficacy on personal norms and subjective norms

From the interdisciplinary perspective of the Theory of Planned Behavior and the Norm Activation Model (Valizadeh et al., 2026; Fauzi et al., 2024), outcome efficacy – defined as the strength of an individual’s belief in whether their behavior can effectively facilitate problem-solving (Hamann et al., 2024; van Valkengoed et al., 2022) – plays a pivotal role in silver-age teachers’ decision-making regarding voluntary teaching participation (Steg and De Groot, 2010; Hamann et al., 2024). For silver-age teachers with decades of experience in teaching, research and administration, their professional backgrounds and career experiences make them attach particular importance to the practical effectiveness of their actions. When they form a well-grounded assessment that their voluntary teaching efforts can effectively improve curriculum quality in recipient schools, foster the professional development of young teachers, or substantially advance disciplinary construction, such positive expectations of behavioral outcomes will significantly strengthen their moral sense of value and responsibility to contribute their residual capacity, thereby directly enhancing the salience of personal norms (Han, 2014; Goetting and Becker, 2025). Conversely, if teachers perceive their efforts as unlikely to generate substantive impacts, their moral responsibility may be weakened by low efficacy (Zhu and Lu, 2025; Masood et al., 2024), even when they recognize the severity of educational inequity.

Meanwhile, outcome efficacy also influences the formation of subjective norms through a social identification mechanism (Ajzen, 1991; Monik and Parzuchowski, 2024). When silver-age teachers judge that voluntary teaching can deliver measurable educational and social value, they will reasonably infer that this socially meaningful behavior will gain endorsement from key reference groups – including the understanding and support of family members, policy safeguards and moral recognition from their former institutions, and positive evaluations from peers and public opinion. Such perceived social support based on outcome expectations further reinforces the subjective normative pressure experienced by teachers.

Based on the above analysis, this study proposes the following hypotheses:

H2a: Outcome efficacy has a positive effect on personal norms.

H2b: Outcome efficacy has a positive effect on subjective norms.

### 2.3 Influence of subjective norms on personal norms

In the behavioral decision-making of silver-age teachers participating in voluntary teaching (Zhan et al., 2022), there exists a profound intrinsic connection between subjective norms and personal norms (Van Tonder et al., 2023). According to the internalization mechanism in social psychology (Crocetti et al., 2023), external social expectations can be gradually transformed into individuals’ internal moral standards through value identification (Klein, 2019; Bandura, 2014; Zheng et al., 2022). For silver-age teachers, when they perceive expectations and recognition for participating in voluntary teaching from their families, former employers, educational peers and even the national level, such positive social pressure will, through a continuous internalization process, prompt them to convert the socially advocated responsibility of supporting education in western China into an innate personal mission and moral pursuit.

The Theory of Planned Behavior (Ajzen, 1991; Lee et al., 2023) and the Norm Activation Model (Schwartz, 1977; Majeed et al., 2023) form an organic connection here: subjective norms and personal norms do not exist in isolation (Meng et al., 2022), but establish a dynamic interaction through the internalization mechanism (Ajzen, 2020; Schwartz, 1977; Stern et al., 1999; Fenitra et al., 2023). When silver-age teachers perceive that key reference groups hold a positive attitude toward voluntary teaching behavior, they will unconsciously integrate this attitude into their own value system, thereby strengthening their personal norms of engaging in voluntary teaching. This path has been fully verified in prosocial behavior research. For instance, studies by Van Tonder et al. (2023) and Song et al. (2023) indicate that individuals’ perceived subjective norms can effectively facilitate the formation of their personal norms. In the context of silver-age teachers, support and expectations from the social environment are ultimately transformed into stable voluntary teaching willingness and motivation for sustained participation through this psychological mechanism of normative internalization.

Based on this, this study proposes the following hypothesis:

H3: Subjective norm has a positive effect on personal norm.

### 2.4 Relationship between personal norms and silver-age teachers’ volunteer teaching intention

According to the core proposition of the Norm Activation Model (NAM), successfully activated personal norms directly translate into the motivation and willingness to perform specific prosocial behaviors (Schwartz, 1977; Wang et al., 2024). This mechanism is particularly pronounced among silver-age teachers (He et al., 2025): when they form a strong personal norm of having to participate in voluntary teaching, based on a profound cognition of educational imbalance, positive expectations of voluntary teaching outcomes, and internalization of social expectations, this internally driven moral identification serves as the most direct and stable psychological foundation for their voluntary teaching willingness (Klein, 2019; Narvaez and Lapsley, 2009; Penner, 2002; Gill, 2023; Steward and Hasche, 2024).

Compared with external incentives or social pressure, behavioral motivation driven by personal norms tends to exhibit greater persistence and stability (Steg and De Groot, 2010; Dagadu et al., 2026). This is especially crucial in the context of silver-age teachers (He et al., 2025)—they generally possess a profound educational commitment, extensive teaching experience and a strong sense of social responsibility, and once they regard voluntary teaching as a personal moral obligation, their participation willingness is unlikely to waver due to short-term difficulties or environmental changes. Extensive research has verified the direct impact of personal norms on behavioral willingness in the fields of pro-environmental and charitable prosocial behaviors (Confente and Scarpi, 2021; Song et al., 2023), further supporting the applicability of this path to the silver-age teacher group. Therefore, stimulating and consolidating their personal norms is of pivotal significance in promoting silver-age teachers’ participation in voluntary teaching and their long-term retention. This intrinsic moral motivation is not only a key driver of their initial participation, but also an internal safeguard that sustains their enthusiasm and enables their sustained participation when facing practical challenges.

Based on this, this study proposes the following hypothesis:

H4: Personal norm has a positive effect on silver-age teachers’ willingness to participate in voluntary teaching.

## 3 Materials and methods

### 3.1 Data source

This study adopted Respondent-Driven Sampling (RDS) for sample selection (Raifman et al., 2022; Sosenko and Bramley, 2022; Heckathorn, 1997). The main rationale is that retired university faculty participating in silver-age volunteer teaching represent a small, scattered, and hidden population without a complete public sampling frame, making traditional probability sampling methods such as random sampling and stratified sampling infeasible (Rendleman et al., 2022). This group forms a close social network based on university peers, silver-age programs, and their former institutions, making it suitable for efficient and targeted recruitment through peer referral chains. Meanwhile, respondent-driven sampling (RDS), a probability-approximate sampling method for hidden populations (McFall et al., 2023; Szwarcwald et al., 2024), can achieve asymptotically unbiased samples through seed recruitment and chain referral, meeting the basic requirements of structural equation modeling for sample representativeness and statistical inference. In addition, surveys initiated by peers are more likely to improve participation willingness and data authenticity (Raifman et al., 2022; Sosenko and Bramley, 2022), which aligns with the overall design of the two-wave longitudinal survey and empirical analysis in this study. First, systematic searches were conducted on the official website of the Ministry of Education of the People’s Republic of China using keywords including silver-age teachers and silver-age program, to identify two pivotal policy documents: Notice of the General Office of the Ministry of Education on the Implementation of the College Silver-Age Teachers Support Western China Program for the 2022–2023 Academic Year and Notice of the General Office of the Ministry of Education on the Implementation of the College Silver-Age Teachers Support Western China Program for the 2023–2024 Academic Year. We extracted the complete list of supporting universities listed in the Pilot Pairing Support Relationships annex of the documents, and visited the official websites of these universities one by one. Through channels such as announcements from the Human Resources Department, Retirement Work Office and campus news, we retrieved and recorded public contact information (e.g., email addresses) of eligible silver-age teachers, initially establishing a contact database of target teachers.

Prior to formally launching the RDS sampling, the research team first conducted preliminary surveys and in-depth interviews (Zainal and Barlas, 2022; Johnson et al., 2024). Through semi-structured interviews with 17 silver-age teachers who had volunteer teaching experience, we gained deep insights into the primary considerations, psychological motivations, and practical challenges faced by this group during their decision-making process for volunteer teaching. Interviewees included retirees who participated in the inaugural Silver Age Teachers Support Western China Program and those with long-term service at higher education institutions in northwest China. All interviews were audio-recorded, transcribed, and thematically coded to provide qualitative support for questionnaire design, item revision, and theoretical model construction. To clearly illustrate the correspondence between interview evidence and research variables, see Table 1 for details.

**TABLE 1:** Interview content (excerpt).

Variables	Interview transcript (original words from the teacher)
Problem awareness	Due to the development gap between the eastern and western regions, there remains a basic disparity between inland areas and border regions, where the foundation is relatively weak. We are short of such teachers here, and it is really difficult to recruit young teachers. There is a particularly high level of importance attached to the national common language, and there is a great demand for it.
Outcome efficacy	I didn’t come here just to give you lessons; I came to lead and build up your team. Assist the college in integration efforts, help with the application for doctoral programs, and establish a linguistics museum. Students have won autonomous region awards, national awards, and Challenge Cup awards, and I have also been honored as an Outstanding Instructor. My major is highly needed in that region and aligns perfectly with the teaching support work.
Subjective norms	I received a lot of support around me. My wife went with me, the university was supportive, and my former colleagues cared deeply about me. My wife has been very supportive. After I had been here for a year, she joined me to teach and accompany me. The Ministry of Education issued the quotas, the university mobilized the faculty, and this was in response to the national counterpart support policy.
Personal norms	As an educator, I believe I have a responsibility and sense of mission to contribute to education in western China. I am here to build up the teaching team, address weaknesses, and support disciplinary development, rather than merely giving lectures. Seeing the students grow and the school develop, I feel that my efforts have been worthwhile and meaningful.
Volunteer teaching intention	The service period was extended from half a year to 1 year, and then to multiple years, providing continuous support in the capacity of a distinguished professor. I haven’t returned yet and is still there participating in teaching and disciplinary development. Despite the long distance and inconvenient living conditions, I am willing to stay and continue my work here for a long time.

Based on this, we clearly defined silver-age teachers as retired university faculty members aged 60–75 who have participated in or are currently participating in the Silver-Age Teacher Support Program for Western Regions. Subsequently, the study entered the “seed” teacher selection phase. The research team sent survey invitations via email to a large number of teachers in the contact database, detailing the research objectives, content, and participation methods. A total of 97 invitation emails were sent. After multiple rounds of communication and eligibility screening, 13 teachers ultimately confirmed that they fully met the study’s definition and formally committed to serving as “seed” teachers. They pledged to participate in the entire research process and take responsibility for recruiting peers in subsequent stages.

On this basis, the official RDS procedure was launched: each of the 13 seed teachers forwarded the research invitation to three eligible target peers, and newly recruited participants continued to recruit their peers in the same manner, gradually expanding the sample size. Considering the potential time lag in the relationships between variables and to control for common method bias, this study adopted a two-wave longitudinal survey design for data collection (Ployhart and Vandenberg, 2010; Podsakoff et al., 2012, 2024; So et al., 2025). The fieldwork of this study was formally conducted from September 2025 to January 2026. First, the questionnaire was designed and revised through a pre-survey and in-depth interviews. In the formal survey stage, informed consent was obtained and documented for every participant throughout the process. Specifically, on the prominent first page of the Wenjuanxing electronic questionnaire, participants were fully informed in writing of the research purpose, content, data usage, confidentiality principles, voluntary participation, and the right to withdraw at any time. All silver-age teachers were required to actively click “I have read and provided informed consent” before proceeding to complete the questionnaire. Meanwhile, the research ethical guidelines and informed items were stated in writing in the invitation emails for seed teacher recruitment and peer referrals, ensuring that participants took part in the survey with full knowledge and free will, with personal privacy and data security protected throughout. Subsequently, a two-wave longitudinal questionnaire survey was carried out using Respondent-Driven Sampling (RDS) from October 2025 to December 2025, with a 2-week interval between the two waves. All data collection and cleaning were completed in January 2026. The two questionnaire surveys were separated by half a month: the first wave mainly measured problem awareness, outcome efficacy and demographic variables, and the second wave measured subjective norm, personal norm and voluntary teaching willingness. To protect privacy and achieve cross-wave questionnaire matching, participating teachers were required to provide the last four digits of their mobile phone numbers as the unique identification code in both surveys. To thank teachers for their participation and improve the response rate, a small electronic gift worth approximately 3 US dollars was provided as a token of gratitude upon completion of each questionnaire. This design can effectively disperse situational interference, weaken the impact of temporary emotions, and reduce the memory anchoring effect of respondents in continuous answering, thereby improving data quality and research validity (Podsakoff et al., 2003; Bozionelos and Simmering, 2022).

The questionnaire of this study was generated as a link via the Wenjuanxing platform, and forwarded layer by layer to participating silver-age teachers through the referral chains in the RDS procedure. The questionnaire content covered problem awareness, outcome efficacy, personal norm, subjective norm, voluntary teaching willingness and demographic variables. Ultimately, a total of 351 valid questionnaires were recovered in this study.

### 3.2 Measurements

To ensure the accuracy and applicability of the scales in the Chinese cultural context, this study adopted a standard back-translation procedure for all adopted foreign scales (Brislin, 1970; Kowal, 2024). First, two bilingual researchers proficient in both Chinese and English independently translated the original scales into Chinese. Subsequently, an English professional teacher who had no prior exposure to the original scales was invited to back-translate the Chinese version back into English. The research team compared the back-translated version with the original scales, conducted multiple rounds of discussion and revision on items with semantic deviations, and finally finalized the Chinese measurement items for all scales.

#### 3.2.1 Subjective norm

Comprising three items, this scale was adapted from Yadav and Pathak (2016) and Van Tonder et al. (2023) to measure the social support pressure perceived by silver-age teachers from three dimensions: family, colleagues, and former institution. Sample items include “My family thinks it is a good thing for me to participate in the silver-age voluntary teaching program,” “Most of my colleagues believe I should participate in the silver-age voluntary teaching program.”, and “My former institution expects me to participate in the silver-age voluntary teaching program.”

#### 3.2.2 Personal norm

Consisting of five items, this scale was adapted from Stern et al. (1999) and Song et al. (2023), focusing on silver-age teachers’ moral responsibility and value identification. Sample items include “I feel a responsibility to contribute to improving the educational situation in western China” and “I would feel guilty if I did not participate in the silver-age voluntary teaching program.”

#### 3.2.3 Voluntary teaching intention

Including four items, this scale was adapted from existing scale designs to measure willingness from three dimensions: participation willingness, sustained willingness, and recommendation willingness (Rausch and Kopplin, 2021; Maričić et al., 2025). Sample items include “I am willing to participate in the silver-age voluntary teaching program,” “I intend to continue participating in the silver-age voluntary teaching program in the future,” and “I am willing to recommend the silver-age voluntary teaching program to relatives and friends.”

#### 3.2.4 Problem awareness

With three items, this scale was adapted from Munerah et al. (2021) to measure silver-age teachers’ cognitive level of educational imbalance in western China. Sample items include “Unbalanced higher education has an adverse impact on the development of western China” and “Unbalanced higher education seriously affects economic and social development.”

#### 3.2.5 Outcome efficacy

Comprising five items, this scale was adapted from Steg and De Groot (2010) and Li et al. (2024) to assess silver-age teachers’ expectations of the effectiveness of voluntary teaching behaviors. Sample items include “I believe that publicity and advocacy activities can promote the popularization and implementation of the silver-age voluntary teaching program” and “I believe that establishing a management organization for silver-age voluntary teaching in my former institution is worthy of promotion.”

## 4 Results

### 4.1 Descriptive statistics

Demographic variables revealed the following characteristics of the surveyed silver-age teachers: In terms of gender, males accounted for 46.44% and females 53.56%. In terms of age distribution, teachers aged 61–65 years constituted the largest group at 46.44%, followed by those aged 66–70 years at 28.21%. Teachers aged 55–60 years (younger retirees) accounted for only 18.8%, and those aged over 70 years made up 6.55%. Regarding retirement income, 42.74% of the respondents had a monthly income of 5,000–7,000 RMB, 35.9% had 7,000–9,000 RMB, 11.97% had 9,000–10,000 RMB, and 9.40% had more than 10,000 RMB. In terms of health status, 76.92% of the surveyed silver-age teachers had no underlying diseases, while 23.08% suffered from chronic diseases such as hypertension and diabetes. Overall, the demographic profile demonstrates that the research subjects in this study are highly representative of the target population.

### 4.2 Common method bias test

As all data in this study were collected through questionnaires, the Harman one-factor test (Howard and Henderson, 2023) was employed to avoid interference from common method bias. An unrotated exploratory factor analysis was performed on all observed items of the variables. The results showed that a total of five common factors with eigenvalues greater than one were extracted, consistent with the preset theoretical dimensions. The variance explanation rate of the first common factor was 39.047%, below the 50% critical threshold (Podsakoff et al., 2003; Huang, 2023). Meanwhile, this study adopted a two-wave separated measurement design to further reduce the risk of method bias. The results indicated that there was no severe common method bias in this study, and the data quality met the requirements for subsequent analysis. Detailed results are shown in Table 2.

**TABLE 2:** Common method bias test.

Factor	Eigen value	Variance explained (%)	Cumulative variance explained (%)
1	7.809	39.047	39.047
2	2.008	10.039	49.086
3	1.681	8.403	57.490
4	1.414	7.068	64.558
5	1.367	6.837	71.395

### 4.3 Exploratory factor analysis

To verify the rationality of the scale structure and the accuracy of item attribution, this study conducted an exploratory factor analysis (EFA) (Howard and Henderson, 2023) on the observed items of the five core latent variables: problem awareness, outcome efficacy, subjective norm, personal norm, and voluntary teaching intention. Prior to the analysis, the Kaiser-Meyer-Olkin (KMO) test (Dalawi et al., 2025) and Bartlett’s test of sphericity (Alliouche and Kouba, 2023) were performed to assess the suitability of the data for factor analysis. The results showed a KMO value of 0.914 (>0.7) and a χ^2^ value of 3743.672 for Bartlett’s test of sphericity (*p* < 0.001), indicating significant correlations among the items and confirming the data was suitable for EFA.

In the analysis process, principal component analysis (PCA) (Elhaik, 2022) was adopted to extract common factors, and varimax rotation (Nguyen and Waller, 2023) was applied for factor rotation to clarify the factor loading attribution of each item. Common factors were extracted based on the criterion of eigenvalues greater than one, and a total of five common factors were finally obtained, which were fully consistent with the preset theoretical dimensions. The cumulative variance explained by the five common factors reached 71.40%, demonstrating the scale had good construct validity and could effectively explain most information of the observed variables. The factor loadings of all observed items on their corresponding latent variables were greater than 0.7 with no cross-loading, indicating good convergent validity of the items for measuring the latent variables. Meanwhile, the Cronbach’s α coefficients of all latent variables were greater than 0.8 (see Table 2 for detailed results), which verified the scale had excellent internal consistency reliability and reliable measurement results (Zhang et al., 2022). The detailed results of the factor analysis are presented in Table 3.

**TABLE 3:** Exploratory factor analysis of influencing factors on silver-age teachers’ willingness to renew employment.

Variables	Research constructs and observable variables	Factor loadings	Eigen root	Cumulative explained variance (%)	Chronbach’s α
Subjective norms	SN1: My family believes that my participation in the Silver Age Volunteer Teaching Program is a good thing.	0.807	2.218	71.40	0.814
	SN2: Most of my colleagues believe I should participate in the Silver Age Volunteer Teaching Program.	0.783			
	SN3: My organization would like me to participate in the Silver Age Volunteer Teaching Program.	0.800			
Personal norms	PN1: I feel it is my responsibility to contribute to improving the current state of education in the western regions.	0.765	3.455	34.63	0.883
	PN2: I believe I should strive to optimize the current state of education in the western regions.	0.757			
	PN3: I believe everyone has a responsibility to consider the impact on the current state of education in western regions when deciding whether to participate in the Silver Age Volunteer Teaching Program.	0.791			
	PN4: My values will drive me to choose to participate in the Silver Age Volunteer Teaching Program.	0.787			
	PN5: Not participating in the Silver Age Volunteer Teaching Program makes me feel guilty.	0.796			
Volunteer teaching intention	VTI1: I am happy to participate in the Silver Age Volunteer Teaching Program.	0.780	2.878	49.01	0.865
	VTI2: I plan to continue participating in the Silver Age Volunteer Teaching Program in the future.	0.790			
	VTI3: I will likely participate in the Silver Age Volunteer Teaching Program in the future.	0.777			
	VTI4: I would gladly recommend the Silver Age Volunteer Teaching Program to my family and friends.	0.817			
Problem awareness	PA1: The imbalance in higher education has adversely affected the development of the western regions.	0.831	2.259	60.31	0.840
	PA2: Imbalanced higher education, due to inadequate faculty resources, leads to subpar educational quality.	0.789			
	PA3: The imbalance in higher education severely hampers economic and social development.	0.793			
Outcome efficacy	OE1: I believe we can promote participation in the Silver Age Program through activities such as publicity campaigns and outreach events.	0.776	3.470	17.35	0.891
	OE2: It is meaningful to promote the Silver Age Volunteer Teaching Program through publicity campaigns and outreach activities.	0.793			
	OE3: I believe that promotional activities such as publicity campaigns can help advance the promotion and implementation of the Silver Age Volunteer Teaching Program.	0.751			
	OE4: I believe organizing activities such as visits, research trips, and field trips to promote Silver Age volunteer teaching will be effective.	0.770			
	OE5: I believe establishing a management organization for the Silver Age Volunteer Teaching Initiative at my school is a worthwhile endeavor.	0.774			

### 4.4 Confirmatory factor analysis

To further examine the structural stability and measurement quality of the scale, this study employed confirmatory factor analysis (CFA) using AMOS 24.0 (Yücel and Cengiz, 2025) to evaluate the five-factor measurement model comprising problem perception, outcome efficacy, subjective norms, personal norms, and volunteer teaching intention. Maximum likelihood estimation was utilized to assess model fit, convergent validity, and discriminant validity. Among the model fit indices,χ^2^/df = 1.526 (<3), GFI = 0.952 (>0.9), RMSEA = 0.039 (<0.08), CFI = 0.976 (>0.9), all meeting statistical criteria. This indicates good model-data fit and alignment with the theoretical framework. Regarding convergent validity, observed item factor loadings ranged from 0.712 to 0.856 (all > 0.7), with *t*-statistics significant at *p* < 0.001. The composite reliability (CR) of latent variables ranged from 0.758 to 0.894 (>0.7), and the average variance extracted (AVE) ranged from 0.597 to 0.647 (>0.5), meeting the criteria for convergent validity. Regarding discriminant validity, the square root of each latent variable’s AVE exceeded its correlation coefficients (Gong et al., 2024) with other variables, indicating clear latent variable boundaries and no dimension confusion. In summary, the measurement model in this study met validity standards and can be used for subsequent path analysis in structural equation modeling. Specific results are shown in Table 4.

**TABLE 4:** Exploratory factor analysis of influencing factor.

Latent variable	Observed variable	Standardized factor loading	*T*-statistic	R^2^	CR	AVE
Subjective norms	SN1	0.779***	–	0.607	0.758	0.635
	SN2	0.766***	13.135	0.586		
	SN3	0.768***	13.167	0.590		
Personal norms	PN1	0.768***	–	0.590	0.894	0.607
	PN2	0.779***	14.782	0.606		
	PN3	0.780***	14.823	0.609		
	PN4	0.787***	14.962	0.620		
	PN5	0.762***	14.436	0.581		
Volunteer teaching intention	VTI1	0.754***	–	0.568	0.846	0.626
	VTI2	0.780***	14.234	0.608		
	VTI3	0.791***	14.431	0.625		
	VTI4	0.817***	14.892	0.668		
Problem awareness	PA1	0.790***	–	0.624	0.762	0.647
	PA2	0.763***	14.111	0.582		
	PA3	0.840***	15.147	0.706		
Outcome efficacy	OE1	0.798***	–	0.637	0.892	0.597
	OE2	0.792***	16.004	0.628		
	OE3	0.779***	15.683	0.607		
	OE4	0.787***	15.886	0.620		
	OE5	0.784***	15.811	0.615		

*P < 0.1; **p < 0.05; ***p < 0.01.

#### 4.5 Correlation analysis

Building upon the validation of the measurement model’s reliability and validity, it is necessary to further clarify the interrelationships and conceptual boundaries among the latent variables of problem perception, outcome efficacy, subjective norms, personal norms, and volunteer teaching intention. Correlation coefficients (Gong et al., 2024), as core indicators measuring the strength of linear relationships between variables, provide an intuitive reflection of the association degree among latent variables. Combined with comparative analysis using the square root of the average variance extracted (AVE), they constitute a critical component of discriminant validity testing (Wang et al., 2023). The following latent variable correlation matrix presents the associative characteristics among variables and verifies their distinctiveness, laying the foundation for subsequent path hypothesis testing in the structural model. The correlation analysis results indicate significant positive correlations among all latent variables, with correlation coefficients ranging from 0.346 to 0.487. This suggests moderate correlations between variables, reflecting the intrinsic connections among theoretical constructs without exhibiting high overlap. These findings preliminarily support the validity of the research hypotheses. Specific results are shown in Table 5.

**TABLE 5:** Correlation analysis.

Variables	SN	PN	PW	PA	OE
SN	1	–	–	–	–
PN	0.418**	1	–	–	–
VTI	0.346**	0.410**	1	–	–
PA	0.370**	0.419**	0.415**	1	–
OE	0.458**	0.438**	0.487**	0.469**	1

*P < 0.1; **p < 0.05; ***p < 0.01.

#### 4.6 Hypothesis testing

**FIGURE 1:**
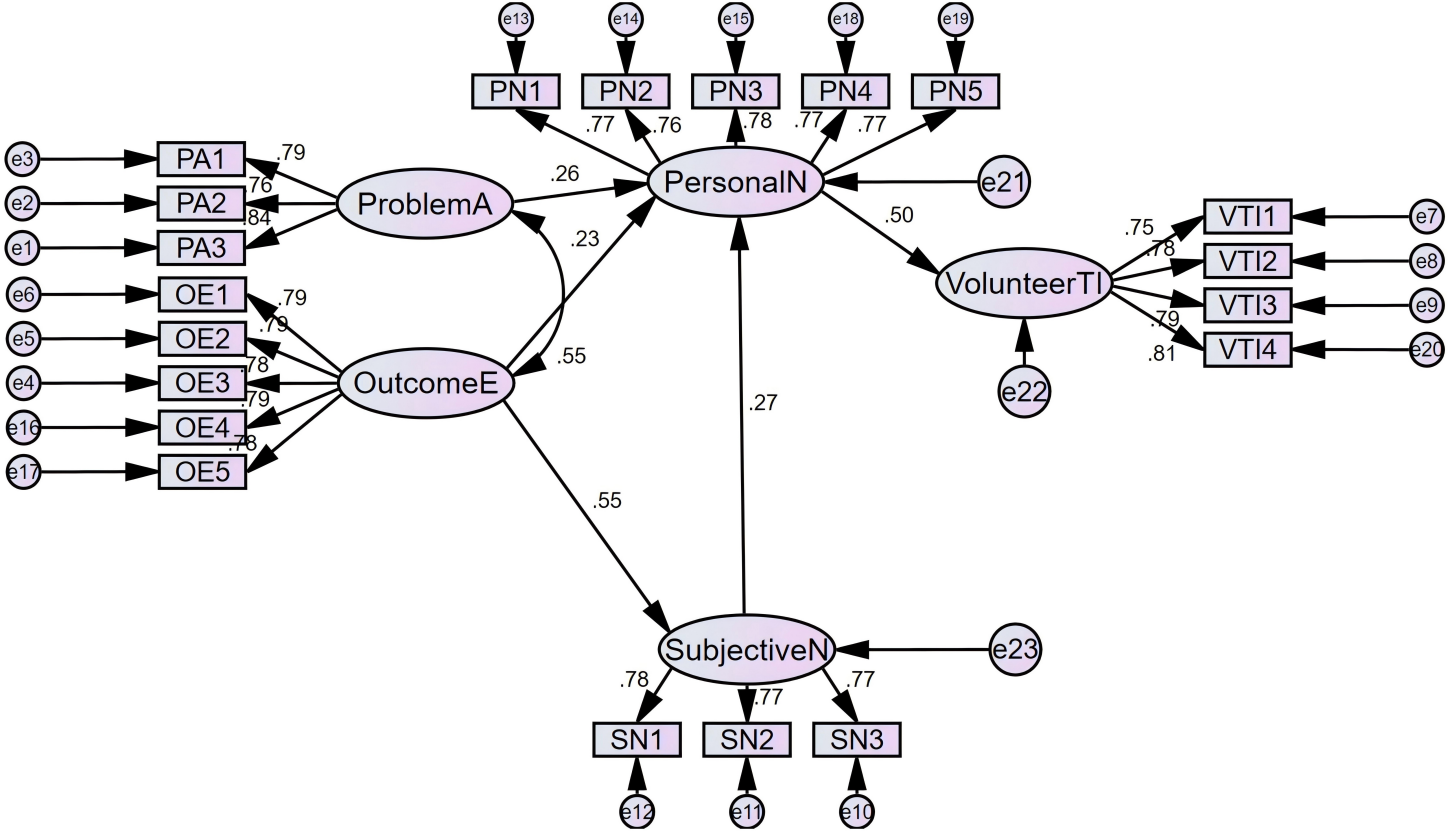
Path diagram of the structural equation model.

**FIGURE 2:**
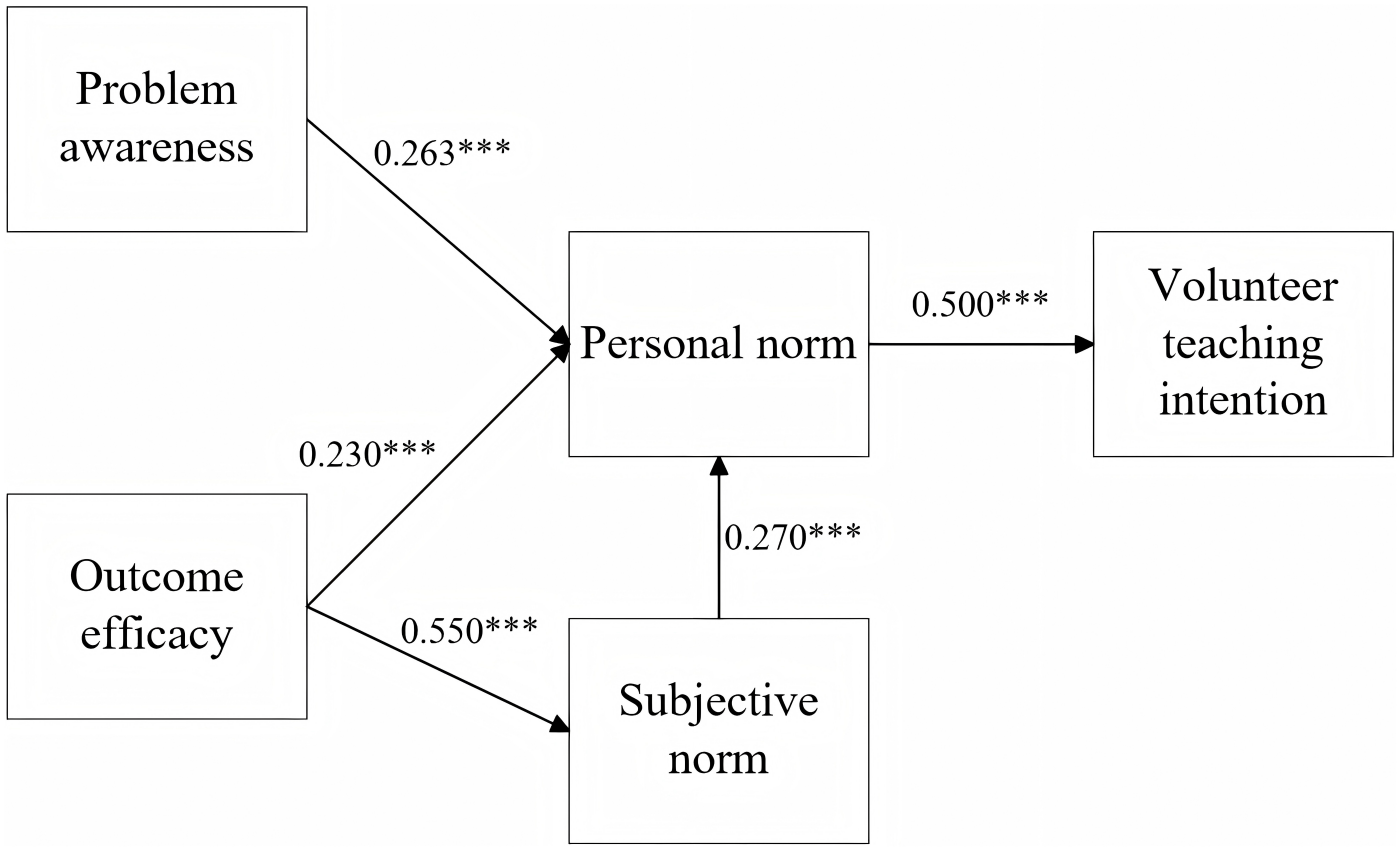
Model validation results.

The core value of structural equation modeling (SEM) (Zyphur et al., 2023) lies in verifying the hypotheses of causal relationships among latent variables in a preset theoretical model. Based on the previously constructed theoretical framework of external cognition – normative internalization – behavioral willingness, this study proposed five research hypotheses regarding the path relationships of latent variables. After confirming the fitness of the measurement model, path analysis was conducted on the structural model using the maximum likelihood estimation method. Standardized path coefficients were used to reflect the intensity of influencing effects, and *p*-values were adopted to judge path significance, so as to verify the validity of the direct and indirect influence mechanisms of different variables on silver-age teachers’ voluntary teaching intention, thereby providing core empirical support for the research conclusions. Detailed results are presented in Figure 1 and Table 6. The path coefficient diagram is shown in Figure 2.

**TABLE 6:** Hypothesis testing results.

Hypothesis	Standardized path coefficient	*P*	Test results
H1	0.263***	0.000	Verified
H2a	0.230***	0.001	Verified
H2b	0.550***	0.000	Verified
H3	0.270***	0.000	Verified
H4	0.500***	0.000	Verified

*P < 0.1; **p < 0.05; ***p < 0.01.

## 5 Discussion

Based on an integrated framework of the Norm Activation Model (NAM) (Nketiah et al., 2022) and the Theory of Planned Behavior (TPB) (Lee et al., 2023), this study systematically explored the internal psychological mechanisms influencing silver-age teachers’ voluntary teaching intention through an empirical survey of 351 college silver-age teachers. The results of structural equation modeling (SEM) (Cheung et al., 2024) supported all research hypotheses, confirming the core mediating role of personal norm in the formation of silver-age teachers’ voluntary teaching intention, as well as the synergistic driving paths of problem awareness, outcome efficacy and subjective norm. This discussion draws on existing literature to elaborate the core findings, while adopting a critical perspective to deeply examine the applicable boundaries, alternative explanations, and broader theoretical implications of the results.

This study verified that personal norm is the most direct and stable predictor of silver-age teachers’ voluntary teaching intention (β = 0.500). This finding is highly consistent with the core proposition of NAM that activated moral responsibility serves as the most immediate psychological driver of prosocial behavior (Schwartz, 1977; Surira et al., 2025). From a critical perspective, the dominant effect of personal norms observed in this study still has other alternative explanations. The volunteer teaching intention of silver-age teachers is not driven solely by moral responsibility; practical conditions such as health status, leisure time, institutional support, regional attachment, and social reputation may also play important roles. Therefore, the norm activation mechanism should be regarded as a key motivation rather than the sole motivation. Compared with the general elderly group, silver-age teachers’ personal norm is more deeply rooted in the professional identity and social responsibility formed during their long educational careers. Such responsibility elevates their prosocial behavior beyond ordinary altruistic motivation to become a continuation and sublimation of professional mission. This finding echoes the generativity theory proposed by Erikson (1963), which states that individuals in late adulthood derive life meaning by guiding the next generation, and highly educated elderly groups tend to realize self-worth through knowledge inheritance (Wang et al., 2026). In this study, silver-age teachers regard voluntary teaching as a continuation of educators’ mission, and this role identity strengthens the predictive power of personal norm on behavioral intention. Meanwhile, it should be noted that there exist boundary conditions: this mechanism is more applicable to highly educated retired teachers with strong educational sentiment and professional identity, and its explanatory power will decrease significantly for elderly groups with limited health or weak professional connection. The results also expand the application boundary of NAM in the elderly group, revealing the unique motivational structure of silver-age teachers’ prosocial behavior—their behavioral decisions are driven more by the sense of continuity of professional identity and the pursuit of intrinsic value, rather than mere external incentives (Atchley, 1989; Omoto et al., 2000; Penner and Finkelstein, 1998; Galkutė and Herrera, 2025).

By verifying multiple paths through which problem awareness, outcome efficacy and subjective norm influence voluntary teaching intention via personal norm, this study uncovered the complex psychological mechanism underlying the formation of silver-age teachers’ behavioral intention. This multi-path model not only confirms the basic framework of NAM, but also enriches our understanding of the motivation for elderly prosocial behavior. The path of problem awareness influencing intention through personal norm (H1) validates the classic cognition-behavior pathway. This finding aligns with conclusions from environmental psychology research on risk perception driving pro-environmental behavior (van Valkengoed et al., 2022; Van der Linden, 2015, 2021; Brosch and Steg, 2021). Nevertheless, in the context of silver-age teachers, the content of problem awareness exhibits distinct occupational specificity—cognition of educational imbalance triggers a specific sense of responsibility as insiders of the education system. This is consistent with the findings of Bouman et al. (2021). Supporting this view, Wang et al. (2024), in their reexamination of consumers’ willingness to stay at green hotels through the lens of social identity theory, value-belief-norm theory, and the theory of planned behavior, also confirmed that problem awareness closely tied to individual identity can effectively activate personal norms. Meanwhile, it should be noted that as a boundary condition, the strength of this pathway may be significantly reduced in non-educational volunteer services or general elderly public welfare participation scenarios. The dual-path effect of outcome efficacy (H2a, H2b) reveals the interactive influence of rational cognition and emotional motivation. On the one hand, outcome efficacy influences intention by strengthening personal norm, supporting the viewpoint of Steg and De Groot (2010) but also with Hamann et al. (2024). On the other hand, the significant impact of outcome efficacy on subjective norm confirms the argument in Bandura’s (2001) social cognitive theory that efficacy expectations shape social judgments. Echoing this theoretical inference, Ellis and Helaire (2023) further verified the inherent correlation between self-efficacy and subjective norms in the framework of the theory of planned behavior. This dual-mechanism effect indicates that silver-age teachers’ decisions are jointly influenced by rational calculation and emotional drive, reflecting the complexity of decision-making among highly educated elderly groups.

The path of subjective norm influencing intention through personal norm (H3) reveals the internalization process of social environmental factors on individual motivation. This finding is consistent with the perspective of external regulation internalization in self-determination theory (Ryan and Deci, 2020; Gagné and Deci, 2005; Lavergne et al., 2010; Tagkaloglou and Kasser, 2018; Kwon and Sonday, 2026). For silver-age teachers who value social evaluation, support from key reference groups not only reduces participation concerns, but also strengthens intrinsic motivation through the pathway of social recognition → self-identification. However, the effect of subjective norms has clear boundaries: its influence intensity varies with age, health status, regional culture and policy environment. Moreover, excessive external expectations may turn into normative pressure, which in turn reduces long-term participation intention. Notably, the mediating path of outcome efficacy → subjective norm → personal norm identified in this study reflects the dynamic interaction between social environment and individual cognition. This path aligns with the goal-framing theory proposed by Lindenberg and Steg (2007) which holds that individuals strengthen their normative goals when perceiving that behavior can generate positive social evaluation, thereby influencing behavioral intention. This logical relationship is further corroborated by Pastor et al. (2024), who demonstrated that subjective norm serves as a critical antecedent linking social responsibility perception to the formation and internalization of individual prosocial normative cognition. In the context of silver-age teachers, cognition of the social value of voluntary teaching ultimately promotes the internalization of personal norm by enhancing subjective normative pressure. Meanwhile, it should be pointed out that this pathway is not fully universal and becomes more prominent only under the premise that silver-age teachers value professional contribution, social value and professional prestige. Overall, this study not only deepens the explanatory power of the integrated NAM-TPB framework (Xu et al., 2024) but also provides more contextualized theoretical implications: the volunteer teaching behavior of silver-age teachers does not simply follow the general prosocial behavior model, but is a unique decision-making process driven by the combined effects of professional identity, intergenerational inheritance, value realization and institutional support, which can offer a more precise theoretical reference for research on active aging, silver-age talent development and educational equity.

The main contributions of this study lie in two key dimensions: theoretical enrichment and methodological advancement. Theoretically, taking the volunteer teaching intention of university silver-age teachers as the research object, this study conducts context-specific integration and empirical verification of the Norm Activation Model (NAM) (Valizadeh et al., 2026; Fauzi et al., 2024) and the Theory of Planned Behavior (TPB) (Lee et al., 2023), further enriching the theoretical framework for explaining the participation motivation of silver-age teachers. This study constructs an integrated model encompassing problem perception, outcome efficacy, subjective norms, personal norms, and volunteer teaching intention, identifies and verifies the core mediating role of personal norms in the formation of volunteer teaching intention, and systematically reveals the psychological transmission pathway of “external cognition–norm internalization–behavioral intention,” providing a more refined mechanistic explanation for understanding silver-age teachers’ volunteer teaching motivation. Specifically, the incremental contributions of this study are reflected in three aspects: first, introducing outcome efficacy into the Norm Activation Model and empirically testing the facilitating effect of outcome expectation on the internalization of moral responsibility, offering supplementary evidence for motivational research on elderly prosocial behavior; second, highlighting the mediating role of personal norms in the Theory of Planned Behavior, clarifying that social norms can stably enhance volunteer teaching intention only through internal moral identification, further improving the explanatory chain of normative motivation transforming into behavioral intention; third, based on the professional characteristics of silver-age teachers, revealing the strengthening effect of educational mission on personal norms, which is more consistent with the value pursuit and behavioral logic of this group and makes theoretical interpretation more context-specific. Methodologically, targeting the specific group of silver-age teachers, this study adopts Respondent-Driven Sampling (RDS) (Raifman et al., 2022; Sosenko and Bramley, 2022) to obtain valid samples, and conducts rigorous empirical tests combining two-wave longitudinal design, exploratory and confirmatory factor analysis, and structural equation modeling, which improves the reliability of the research findings and provides replicable methodological practices for similar studies.

This study initially revealed the key psychological mechanisms influencing silver-age teachers’ voluntary teaching intention through SEM, but there are several noteworthy limitations. First, although a two-wave data collection design was adopted, variable measurement was still concentrated within a relatively short time window, making it difficult to fully establish causal relationships among variables (Yu et al., 2026). Future research needs to adopt longer-term longitudinal tracking design to clarify causal timing. Second, the research sample was mainly derived from retired teachers of key universities participating in the College Silver-Age Teachers Support Western China Program, and the generalizability of the conclusions to retired teachers from ordinary colleges, primary and secondary schools needs further verification (Wang et al., 2022). Third, the existing theoretical model has not incorporated potential important variables such as institutional trust and regional cultural differences, nor explored the cross-level moderating effects of contextual factors such as school support policies or regional development levels (Xu and Zhang, 2023). Finally, the study focused on the measurement of behavioral intention rather than actual voluntary teaching behavior. Future research needs to further track the specific pathways of transformation from intention to actual behavior and the potential hindering factors involved (Feil et al., 2023). These limitations point to clear directions for subsequent research, including expanding the theoretical framework, adopting multilevel modeling methods, and conducting long-term follow-up surveys, so as to more comprehensively and deeply understand the decision-making process and behavioral patterns of silver-age teachers.

## 6 Conclusion

Based on the integration of NAM and TPB and empirical analysis of 351 college silver-age teachers, this study draws the following core conclusions: (1) Personal norm is the most direct and critical psychological factor driving silver-age teachers’ voluntary teaching intention. SEM path analysis shows that personal norm has a significant positive direct impact on voluntary teaching intention, serving as the core bridge connecting external cognition and behavioral intention. (2) Problem awareness, outcome efficacy and subjective norm all indirectly influence voluntary teaching intention through the mediating role of personal norm, forming a transmission mechanism of external cognition – normative internalization – behavioral intention. Among them, outcome efficacy not only positively affects personal norm, but also significantly strengthens subjective norm. (3) This study verified an integrated psychological motivation model, revealing that silver-age teachers’ voluntary teaching decision is a complex process jointly influenced by rational cognition, moral emotion and social environment, which makes up for the explanatory limitation of a single theoretical model.

Based on the above conclusions, to effectively stimulate and maintain silver-age teachers’ motivation for voluntary teaching, this study proposes the following multi-level recommendations:

(1) At the policy level, efforts should focus on the four core psychological pathways of problem perception, outcome efficacy, subjective norms, and personal norms to build a support system aligned with the formation mechanism of silver-age teachers’ volunteer teaching intention. Policy advocacy and displays of the educational situation in recipient regions should be used to raise silver-age teachers’ awareness of educational resource imbalance; regular feedback channels on volunteer teaching outcomes should be established to strengthen teachers’ perception of the practical value of their teaching; positive support from families, former institutions, recipient schools, and society should be enhanced to improve subjective norms; on this basis, external cognition and social support should be transformed into internal moral responsibility to consolidate and strengthen personal norms, thereby continuously boosting volunteer teaching intention.

(2) Universities and program organizers should optimize the recruitment, training, and service management of silver-age teachers in line with the internal logic of “external cognition–norm internalization–volunteer teaching intention.” During recruitment and pre-service training, information on educational needs in recipient regions should be better communicated to enhance teachers’ problem perception; during volunteer teaching, timely feedback on teaching improvements, student growth, and disciplinary development should be provided to strengthen outcome efficacy; organizational support, honor incentives, and social recognition should be used to foster a positive atmosphere, reinforce subjective norms, and promote the internalization of external norms into personal norms, so as to elevate volunteer teaching intention and sustained participation motivation.

(3) Recipient schools should provide supporting services centered on improving outcome efficacy, strengthening subjective norms, and advancing norm internalization. Timely feedback should be given to silver-age teachers on teaching effectiveness, student development, and disciplinary construction to reinforce their perception of volunteer teaching value; subjective norms should be enhanced through full respect, instructional support, and positive recognition; regular affirmation of value and outcome feedback should be used to turn external norms into teachers’ moral responsibility and mission identity, steadily strengthening personal norms and thereby raising volunteer teaching intention.

## Data Availability

The original contributions presented in this study are included in this article/supplementary material, further inquiries can be directed to the corresponding author.
